# Extracting and modeling geographic information from scientific articles

**DOI:** 10.1371/journal.pone.0244918

**Published:** 2021-01-06

**Authors:** Elise Acheson, Ross S. Purves

**Affiliations:** Department of Geography, University of Zurich, Zurich, Switzerland; University of Wisconsin Madison, UNITED STATES

## Abstract

Scientific articles often contain relevant geographic information such as where field work was performed or where patients were treated. Most often, this information appears in the full-text article contents as a description in natural language including place names, with no accompanying machine-readable geographic metadata. Automatically extracting this geographic information could help conduct meta-analyses, find geographical research gaps, and retrieve articles using spatial search criteria. Research on this problem is still in its infancy, with many works manually processing corpora for locations and few cross-domain studies. In this paper, we develop a fully automatic pipeline to extract and represent relevant locations from scientific articles, applying it to two varied corpora. We obtain good performance, with full pipeline precision of 0.84 for an environmental corpus, and 0.78 for a biomedical corpus. Our results can be visualized as simple global maps, allowing human annotators to both explore corpus patterns in space and triage results for downstream analysis. Future work should not only focus on improving individual pipeline components, but also be informed by user needs derived from the potential spatial analysis and exploration of such corpora.

## Introduction

Geographical information permeates the written world, appearing as place names or place descriptions in texts including news articles, blog posts, social media content, historical documents, and scientific articles. Research on extracting geographical information from text has often focused on news articles [1–3] and social media content [4–6], with surprisingly limited attention being directed towards the increasing number of published scientific articles. Indeed, with each passing year, scientists face an ever-growing stack of scientific articles to sort through, read, understand, and build upon. Many of these articles contain important geographical information: perhaps soil samples were taken from a certain region, patients were treated in a particular hospital, or interviews were conducted in a village or neighborhood. Currently, researchers must manually sift through article contents to identify any relevant locations, a time-consuming process. Furthermore, linking these textual place descriptions to spatial representations (such as point coordinates, a bounding box, or a polygonal region) requires significant additional work and should ideally respect the scale and precision of locations described in the text. Despite discussions about the need to develop and adopt metadata reporting standards for geographic information [7–9], the vast majority of scientific articles continue to be published without any accompanying machine-readable spatial data, though geographic information often appears in the article contents in textual form. The ability to automatically extract and spatially represent this geographic information would enable researchers to organize and find information using not just keywords but also spatial criteria, as is done for other types of text using Geographic Information Retrieval (GIR) techniques [10]. Organizing and visualizing scientific corpora by space would facilitate geographically-aware meta-analyses [11], enable studies to be cross-referenced by location [12, 13], and allow for the discovery of geographical research gaps such as understudied regions in a particular scientific discipline [14, 15].

Though scientific articles have become a frequent object of study for researchers, common research objectives are to analyze and visualize (often large) article collections [16–18], and to extract or summarize specific information from publications through text mining, usually in a particular domain such as biomedical research [19, 20]. On the one hand, many scientific corpus analyses consider geography, but focus on author locations which are easier to extract from articles [17, 21], and on the other hand, many specialized text mining tools go beyond article metadata and into full-text processing, but don’t give special treatment to geographical information. Meanwhile, extracting and representing meaningful geographical locations such as study sites from scientific articles remains a challenging and understudied problem. Most published works on this problem identify relevant locations from text manually [12, 15, 22, 23], and few tackle the problem using a scalable, automatic approach [9, 24, 25]. When automatic approaches are used, they are constrained in their applicability, either by only extracting geographic coordinates [26, 27], by not utilizing the full-text of articles [14], or by performing overly poorly on full-text [9]. Furthermore, the corpora used remain limited both in size and disciplinary focus, potentially limiting the wider applicability of the techniques and findings.

A long-standing related and relevant stream of work that has recently been applied to scientific articles is the detection and disambiguation of place names (toponyms), a task known as toponym recognition and resolution. One recent strand of work has concentrated around an annotated corpus related to phylogeography [28]. This work includes a series of publications [28–31] and a SemEval-2019 task called ‘Toponym Resolution in Scientific Papers’ [32]. However, these research efforts focus on identifying *all* toponym mentions within the main text of an article, rather than a subset of relevant locations representing, for example, where a study was conducted. This means that annotated toponyms in this phylogeography corpus include toponyms listed alongside company locations (for chemicals or products used in a study) as well as toponyms mentioned in the context of scientific background. The present work focuses on a different, albeit related, task: automatically extracting and geographically representing meaningful or relevant locations from scientific articles such as study sites, patient treatment locations, and sample locations. These are almost always a (relatively small) relevant subset of the textual locations or toponyms that appear in the article contents, and thus our task relates more closely to finding the geographic scope of text documents [33–35] than to performing comprehensive toponym resolution on each document [36]. Our goal in this paper is rather to replicate what a human annotator would extract from a scientific article for the purposes of a meta-analysis, or what an author would potentially include as geographical metadata for a submitted article.

Indeed, an important part of processing scientific articles is not only to detect locations, but also to ignore irrelevant locations such as locations in references, locations indicating where a company providing commercial products is based, or locations appearing in expressions such as ‘the Declaration of Helsinki’. The presence of irrelevant place names throughout scientific articles is cited as a major obstacle to automatically extracting study sites using place names in [27] and affected performance and processing decisions in [14, 25]. In our task, each individual place name or toponym mention (which we refer to as location mentions since named locations like hospitals and universities are of interest to us but not necessarily considered to be ‘toponyms’) appearing in text is not equally important, including repeated locations, as long as the correct study locations are captured, as measured through precision and recall.

**Fig 1:**
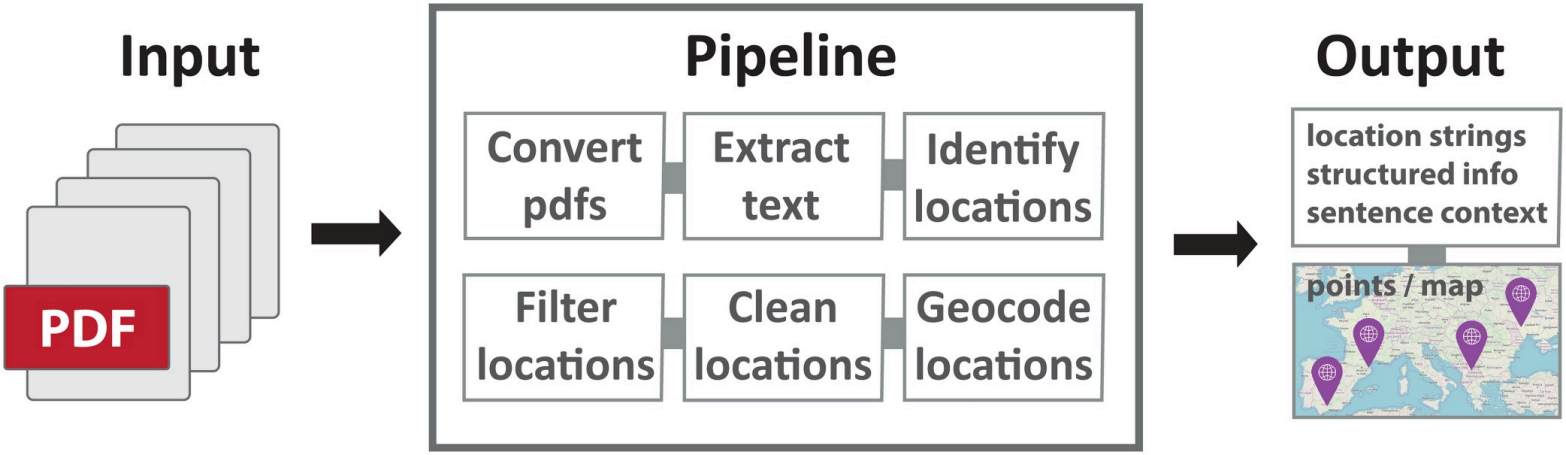
Overview of the processing pipeline. The pipeline starts from scientific article PDFs and outputs extracted locations, with textual and spatial representations.

In this paper, we develop a fully automatic pipeline which starts from a collection of scientific articles and their PDFs and outputs a set of location strings and their sentence context, as well as structured information and a geometric representation for each string (Fig 1). We use two contrasting corpora from two different research domains: 1. a highly spatial ecological research corpus of articles relating to orchards, with most including study site descriptions, and some including maps and coordinates, and 2. a less spatial biomedical corpus of articles on cancer genetics, where many articles fail to report geographical locations at all. Our pipeline combines freely available tools with rule-based processing to extract and represent relevant locations, and aims to minimize domain-customization across our two corpora. We focus on extracting locations from targeted portions of the article, including the title and any methods or study site sections deemed likely to contain relevant locations. We aim to ignore irrelevant locations, such as locations representing where certain scientific products were obtained or manufactured, not only by targeting certain text portions but also through rule-based post-processing of candidate locations. We obtain good performance, with full pipeline precision of 0.84 for the ecological and 0.78 for the biomedical corpus, allowing us to map and discuss the spatial properties of the collections.

## Background

### Extracting geographical information from scientific articles

Automatically identifying place names and their associated spatial language in text is a well-studied problem known most commonly as toponym recognition [37], and is typically the first of several steps required to map or spatially index a corpus [10]. Approaches to toponym recognition (or more broadly, location identification) in scientific articles have thus far mainly consisted of *rule-based* and *gazetteer-based* approaches [37]. A gazetteer-based approach consists of looking up words or sequences of words in a place name database (a gazetteer), where a match indicates a (likely) location. The main downside of this approach is that many common words appear in gazetteers as locations, such as ‘bath’, ‘nice’, and ‘of’, and hence false positives must be limited via post-processing or careful targeting of words to look up. In one example of this approach [24], sentences are first tagged with part-of-speech (POS) labels (such as ‘noun’, ‘adjective’, or ‘noun phrase’), and any noun phrases containing capitalized words are looked up in the GeoNames gazetteer and in Google Maps. A rule-based approach is used in [25] which detects patterns of relevant words, including words found in a gazetteer (likely to be a location), location modifiers (e.g ‘north’), and entity type words (e.g. ‘river’ or ‘mountain’). Good performance is obtained after adding custom pre- and post-processing steps, such as enhancing word lists with geology-specific terms and detecting citations in order to skip them as location candidates.

A commonly used method to identify toponyms in text is to run a Named Entity Recognition (NER) tool over the text and retain the subset of entities which are tagged as locations. However, out-of-the-box NER tools have often been trained mostly on news articles and their performance tends to decrease when texts diverge in form and content from these [38]. An NER tool is considered in [25] for the task of extracting geographical/geological locations in geology articles, but rejected in favor of a rule-based approach due to poor performance. In a series of papers on the aforementioned phylogeography corpus, custom NER tools are developed to identify toponyms, including first using a rule-based approach [28], followed by higher-performing machine learning models using first Conditional Random Fields (CRFs) [30], then bi-directional recurrent neural networks (RNNs) [31]. However, the custom NER tools require re-training on an annotated corpus, as opposed to out-of-the-box tools which can be more readily applied to varied corpora, and no filtering is done to identify only a relevant subset of toponyms/locations.

In this work, we use a pre-trained, freely available NER tool and combine it with rules to deliver as output a subset of relevant locations for each scientific article, such as study sites or patient treatment locations. We focus on extracting these relevant locations by targeted specific portions of the article (pre-NER processing) and by filtering candidate locations to exclude company locations and other irrelevant locations (post-NER processing).

### Geographically representing scientific articles

Once locations have been identified and extracted from an article, a subsequent step is required to convert these textual locations to an explicitly spatial representation. This step is referred to as toponym resolution [39], grounding, or geocoding, and involves both resolving ambiguity (such as, determining whether the string ‘Zürich’ refers to the city of Zürich, the canton of Zürich, or perhaps even Zürich airport) and assigning a geometry to represent the location (such as a latitude, longitude point for the city of Zürich, Switzerland). Geometries are usually obtained by linking the extracted location to a particular gazetteer record which also contains a geometry. In practice, this step can simply consist of querying a *geocoding* service with the location string to get back a ranked list of results, including structured information and a geometry for each, typically a point representation. To aid disambiguation, additional geographical context can be given to most geocoding services, such as a bounding box or country of interest to limit the results, or an augmented string with a containing region such as a state or country. Examples of geocoding services include the Google Geocoding API, OpenStreetMap (OSM) Nominatim, and the GeoNames search webservice.

In previous work dealing with geographic locations in scientific articles, the toponym resolution or geocoding step is sometimes absent, with the focus still largely on developing better methods to identify the locations of interest in text [25, 30]. Furthermore, many of the works which map study sites annotated article collections manually and hence do not perform automatic geocoding [12, 15, 22, 23]. Of the works which perform geocoding automatically, [24] use the relevance-sorted results from both GeoNames and Google Maps and look for a containing country in the same sentence as the location string, while [14] also use the Google Maps API and rely on semi-automatic post-geocoding filtering to limit the number of false matches. In [28], GeoNames search results are disambiguated using a population heuristic (choosing the result with the highest population), a distance heuristic (choosing the result which minimizes the total geographical distance to all other toponyms in the document), and a ‘metadata’ heuristic tailored to their phylogeographic data. In [9], location strings are linked to gazetteer records but no disambiguation is performed, which leads to many false positive matches.

In this work, we use the Google Geocoding API, a high-performing tool, to get structured information and a spatial representation for our extracted location strings. The returned information includes a fully qualified location string, a return type with granularity indications, as well as a latitude and longitude which can be mapped. We programmatically generate maps from these results for each corpus, which gives a visual overview of the overall spatial coverage of the articles.

## Materials and methods

### Corpora

We benefited from the use of two article corpora to work with, which had already been identified as of interest for domain-specific meta-studies:

- **Orchards**: This corpus consists of articles relating to fruit orchards, collected to conduct a meta-analysis on the impact of agricultural practices on biodiversity [40], with an intended focus on orchards in a Mediterranean climate. We obtained an early, minimally-triaged collection of articles to develop our methods. The articles are from a varied list of ecology-related journals, with the top 4 most frequent journals being ‘Environmental Toxicology and Chemistry’, ‘Agroforestry Systems’, ‘Archives of Environmental Contamination and Toxicology’, and ‘Apidologie’.
- **Cancer**: This corpus consists of articles used in the curated cancer genomics database Progenetix (https://progenetix.org), specifically focused on Comparative Genomic Hybridization experiments, alongside Whole Genome/Exome Sequencing studies [41]. As part of data curation, locations are manually extracted for each article, which is currently done by taking the location of the first author, rather than by manually looking through the article contents for locations such as where patient material was obtained. The top 4 most frequent journals for articles in this cancer-genetics-focused collection are: ‘Genes, Chromosomes & Cancer’, ‘Cancer Genetics and Cytogenetics’, ‘Journal of Pathology’, and ‘Oncogene’.

We manually annotated 150 articles in total for the Orchards corpus and 200 for the Cancer corpus (Table 1). The articles were randomly chosen for annotation from a wider set of articles which, for the Cancer corpus, were in the Progenetix database and had a full PDF available, and for the Orchards corpus, had been obtained from targeted keyword searches (as described in [40]) but were not extensively triaged. For each corpora, we set aside 50 randomly sampled articles to use as a test set; our training set consisted of the remaining annotated articles, which we used to develop our processing pipeline, including methods section detection, location extraction, and location geocoding.

**Table 1:** Summary information about the two corpora used.

Corpus		Articles (annotated)		
name	domain	total	train	test
Orchards	ecology	150	100	50
Cancer	biomedical	200	150	50

**Fig 2:**
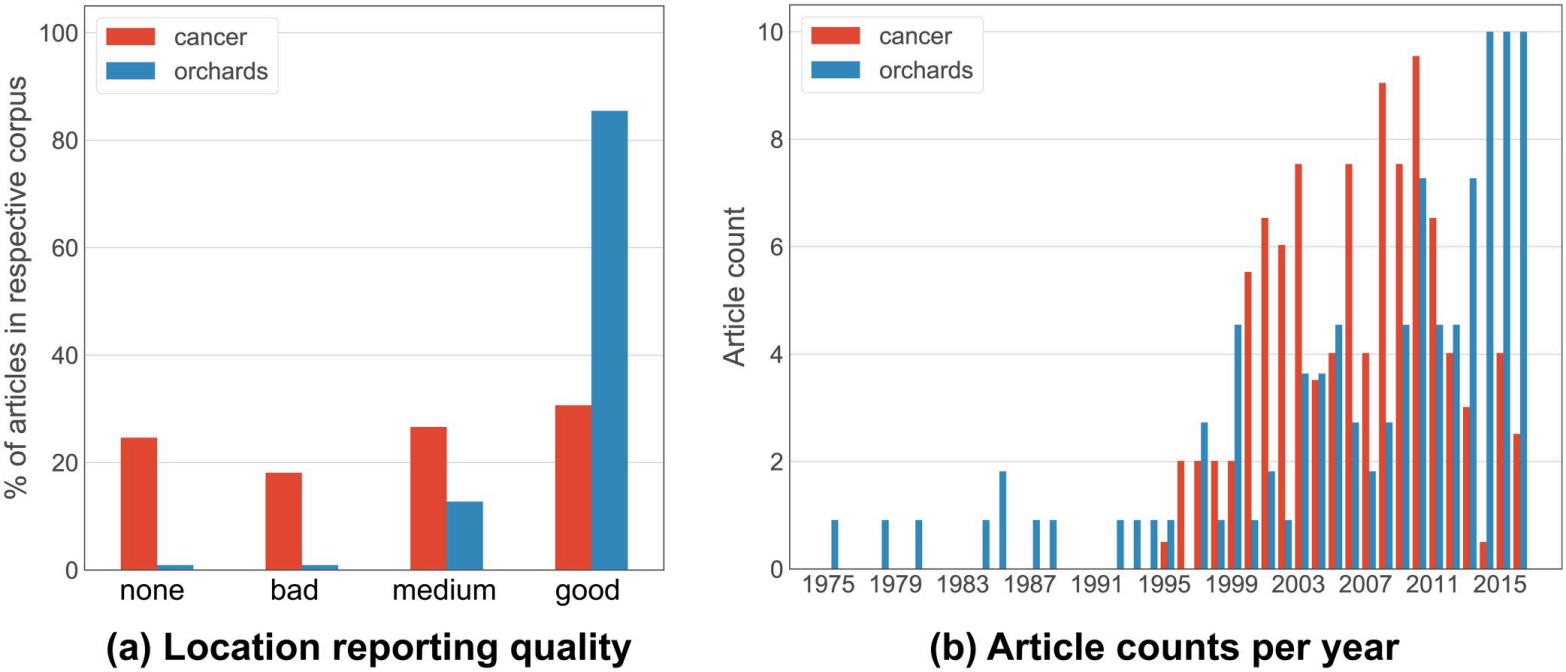
Comparison of the Orchards and Cancer corpora. (a) location reporting quality in the article contents, (b) publication year of the articles. Categories for location reporting quality (a): none: no mention of study/sample location; bad: implicit location info or reference to another paper; medium: study/sample location info like name of institute only and perhaps some locations not mentioned; good: explicit study/sample location info that could probably be extracted and geocoded.

In addition to annotating the ground truth locations which we found in the article contents, we also systematically annotated the quality of the textual location information and, to help develop our methods, where this information was present in the article. Our annotations show that the location reporting quality is varied in the Cancer corpus, but nearly always of high quality in the Orchards corpus (Fig 2(a)); we report further on the location reporting quality over time in S1 Fig. In terms of the year of publication of the articles in our two corpora, it is the Orchards corpus that shows greater variation, with articles spanning the range 1975-2016 (Fig 2(b)); the oldest article in the Cancer corpus by comparison is from 1995, which makes sense considering the corpus’ focus on particular scientific techniques which were only developed in the 1990s.

### Processing pipeline

We now describe the steps of the processing pipeline we applied to the two corpora, followed by any corpus-specific customizations we made to our code. Our code alongside article information is available at https://github.com/eacheson/pyscine. In general, we tried to limit extracted locations to *relevant* locations in two ways: 1. by only looking for locations in targeted portions of the article (pre-NER *Extract text* step) and 2. by filtering identified locations (post-NER *Filter locations* step).

- **Convert PDFs**: The pipeline starts from a set of PDF documents, and converts each document to 1. a plain text file, using pdfminer (https://github.com/pdfminer/pdfminer.six), and to 2. an XML file using CERMINE [42], a Java-based library to extract metadata and contents from scientific article PDFs. Performing two independent file conversions means the pipeline has the possibility to recover from a failed XML conversion or from insufficient headings in the XML file.
- **Extract text**: The next step targets portions of the article contents in which to look for location information; this is done by identifying relevant headings (such as methods or study site sections) using regular expression matching. Matches are found by testing each paragraph beginning in the text files and each heading in the XML files. When a match is found, paragraphs under the matched heading are stored for the next step. At the end of this step, the pipeline continues using only the XML files, unless relevant headings/text were identified in the text files and not in the XML file. The article title identified in the XML file is also separately retained for the further step.
- **Identify locations**: The text portions extracted in the previous step are now processed for locations. First the text is split into paragraphs, normalized (replacing e.g. accented characters with a canonical form), split into sentences and words, and a part-of-speech (POS) tagger is run over each sentence. The text is now ready for NER, which is performed using Stanford NER [43], accessed from the NLTK python library [44] (Stanford NER v3.8.0, NLTK v3.2.5). Stanford NER outperformed the competition on recent NER multi-dataset comparisons [45] and also performed well on location identification more specifically [46]. A 3-class classifier is used which tags each word as one of ‘location’, ‘person’, ‘organization’, or ‘other’ meaning not a named entity. These token (word, tag) combinations from the NER output are processed using custom code which retains sequences of tokens as location candidates for triaging. The goal of this step is high recall, that is, to miss as few true location descriptions as possible. Accordingly, we keep any sequence of words with at least one named entity and include within these sequences words that often appear within a location string, such as ‘in’ or ‘upon’ and two-letter state abbreviations.
- **Filter locations**: The location candidates identified in the previous step are now filtered using rules to remove any candidate that is not deemed a relevant location, including non-locations, suspected company locations, and citations. The rules in this step were developed iteratively on the training set and are based on: tag sequences (e.g. reject candidates with no ‘location’ tags), presence of keep words (e.g. keep candidates with ‘University’ or ‘Institute’), presence of discard words (e.g. reject candidates with ‘Inc’ or ‘GmbH’), and token (tag, word) combinations. The goal of this step is to increase precision, while maintaining good recall. This step produces our final list of identified content locations.
- **Clean locations**: Each content location string retained in the previous step is cleaned of any trailing prepositions or punctuation before the geocoding step.
- **Geocode locations**: Each clean location string is sent to the Google Geocoding API, and the top result is retained (if any results are returned). Each geocode result provides structured location information, including a qualified string representation of the location (such as ‘San Francisco, CA, USA’ for the query ‘San Francisco’), a latitude, and a longitude.

**Fig 3:**
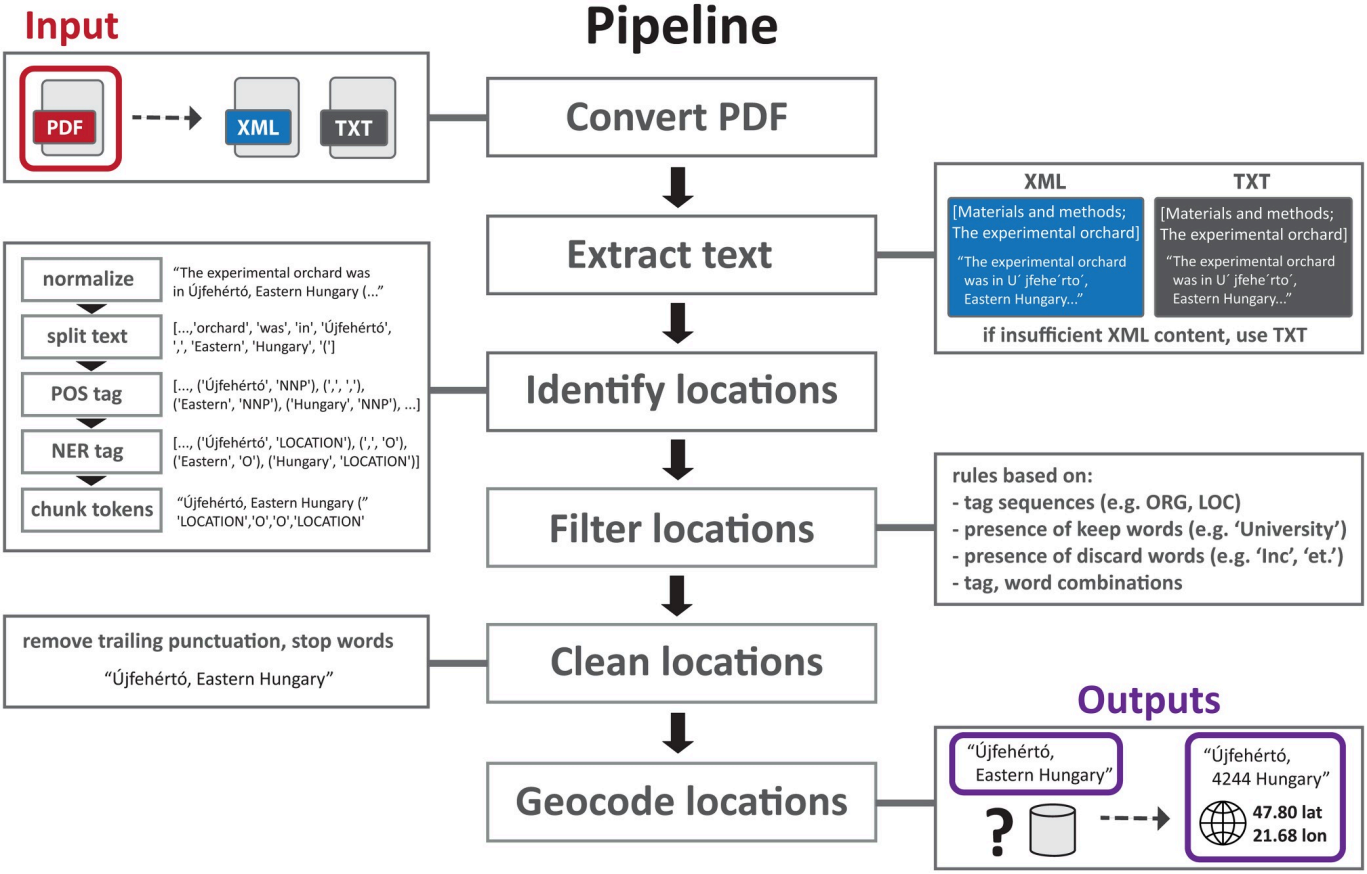
Detailed view of the article processing pipeline. Pipeline illustrated using an example from the Orchards corpus.

The processing pipeline is illustrated in Fig 3. Note that whenever a location candidate is retained, the sentence it was found in is also retained, so that the final output consists not only of identified content location strings and their geocode result information, but also of their sentence context. This not only facilitates our own evaluation, but allows for complex compositional location descriptions (such as ‘30 km from Florence, Italy’) and coordinates appearing in text (such as ‘Florence, Italy (43.77° N, 11.26°E)’) to be retained in our structured output for a human annotator to easily access, as these are typically in the same sentence as a location that our pipeline does retain (such as ‘Florence, Italy’ in both previous examples). We also adapted and ran the coordinate parsing code from [27], but it performed poorly on our data because coordinate strings were often transformed erroneously during conversion from PDFs to plain text and XML files.

We minimally customized our pipeline for either the Orchards or the Cancer corpus. The first and most important customization was in the regular expressions used to detect relevant section headings (*Extract text* step). Relevant headings in the Orchards corpus featured words like ‘region’, ‘area’, and ‘site’, whereas in the Cancer corpus, words indicative of a relevant section heading included ‘patient’, ‘sample’, ‘specimen’, and ‘subject’. The second customization was in the rules used to retain certain sequences of tokens as location candidates (*Identify locations* step). In the Orchards corpus, location strings often contained cardinal direction words (such as ‘east’, ‘southern’, or ‘northeastern’) as well as geographic entity type words (like ‘region’, ‘county’, and ‘park’). We found that including these words in our final location strings had an overall positive effect on the geocoding step, mainly because it tended to keep location words describing the same location together as one string as opposed to two distinct strings (such as ‘Nancy (East of France)’ instead of ‘Nancy’ and ‘France’), giving better context for the geocoding step.

## Results

Our pipeline produced two main outputs: extracted location strings and geocoded results. In addition, evaluation could be performed against two slightly different units: location units or article units. In order to evaluate our two main outputs separately as well as in sequence, we first evaluated our pipeline in 3 stages, using the location unit (Table 2): 1. first, we calculated whether each extracted string was correct (a true positive) or not, giving a value for extraction precision; 2. we then separately evaluated the geocoding using the subset of true positive extracted location strings by calculating how often the geocode result for these strings was correct or incorrect, giving a value for geocoding accuracy; 3. we finally looked at the full set of extracted location strings (true and false positives) and evaluated the final geocode result for each, giving a value for full pipeline precision. This full pipeline evaluation includes several cases where the final result is worse than the individual steps (a correct location string was extracted, but geocoded to the wrong location, a false positive overall), but also a few cases where the full pipeline is better than the individual steps (a wrongly extracted location had no geocode result, resulting in a true negative overall). This is reflected in Table 2, where the full pipeline precision is slightly lower than the extraction precision for both corpora. For the Orchards corpus, we present the results on a subset of articles consisting of studies, rather than review articles, editorials, or articles in popular science magazines. These studies formed between 73-74% of articles in the full minimally-triaged collection, training set, and test set. For results on the complete Orchards corpus, see the S1 Table.

**Table 2:** Results for both corpora, organized according to whether the location unit or article unit was used in evaluation.

	location unit			article unit		
	extraction	geocoding	full pipeline	extraction (weighted)		
corpus	precision	accuracy	precision	precision	recall	F1
Orchards	0.869	0.906	0.842	0.827	0.809	0.818
Cancer	0.810	0.980	0.778	0.740	0.769	0.754

In a second evaluation, we evaluated extraction precision, recall, and *F*1 using the article unit, in order to not give a disproportionate amount of weight to articles with multiple study sites or sample locations. Specifically, we calculated both precision and recall out of a maximum value of 1 for each article, where a precision of 1 meant all extracted location strings were correct, and a recall of 1 meant all ground truth locations (e.g. study sites) were represented in the extracted strings. We then summed these values for an overall precision and overall recall, respectively (Table 2). Precision and recall were combined into one value, *F*1, through their harmonic mean. Any locations extracted from the title were included in this overall pipeline evaluation for the Orchards corpus, as it was determined at the training stage that the titles in this corpus, but not in the Cancer corpus, contained useful locations.

Indeed, in the Orchards test corpus, 19 titles contained a location in the title, whereas in the Cancer test corpus, just one title arguably contained a location, but in adjectival form (i.e. ‘Korean tumours’). We achieved very good performance on title extraction in the Orchards test corpus, with 0.95 for both precision (18/19) and recall (18/19), and hence also *F*1. These locations were often a good overall summary of the study region, but were also fairly often vague regions: in this test set, 6 out of 19 ground truth locations had some inherent vagueness (examples include ‘Southern Russia’, ‘eastern Spain’, ‘European Alps’).

Our results in Table 2 show that generally performance was superior on the Orchards corpus, which is consistent with the superior location reporting quality in that corpus (Fig 2(a)). However, the geocoding accuracy was higher for the Cancer corpus. Though both corpora often had location strings which weren’t fully qualified with a city or country, the Google Geocoding API still mostly gave correct answers for unqualified strings in the Cancer corpus (e.g. ‘Massachusetts General Hospital’, ‘Royal Free Hospital and Medical School’) but not in the Orchards corpus (e.g. ‘Via Emilia’, ‘Dry Creek Vineyard’). Indeed, generally the Orchards corpus featured study sites in lesser known locations outside of cities, whereas the Cancer corpus featured more well-known location names such as cities in Europe and North America, and large hospitals or research Universities.

We systematically classified the errors in our pipeline based on the 3-stage evaluation results (Tables 3 and 4). Only the main source of error for each location unit was recorded and only when the full pipeline result was incorrect did we record an error. NER errors were the most frequent kind of error, followed by not having extracted the paragraph or sentence containing the location string (hence not making it to the NER step). ‘Comma group’ errors occurred when there were multiple, separate locations separated by commas, which our code chunked together as a single qualified location (e.g. ‘Burlington, Cambridge’ where Burlington and Cambridge were separate towns in Canada, instead of Burlington being contained by Cambridge). These comma group errors were all in the Orchards corpus and 3 of them were in one article which listed several countries one after another, something which could be adjusted in code by detecting comma-separated countries.

**Table 3:** Errors in both corpora classified into categories.

	Orchards		Cancer	
error description	count	percent	count	percent
NER error	12	27.3	8	32.0
text portion not extracted	8	18.2	7	28.0
wrong/no geocode result	9	20.5	1	4.0
comma group	7	15.9	0	0.0
candidate filtering error	3	6.8	4	16.0
non-standard headings	3	6.8	0	0.0
other	2	4.5	5	20.0
total	44	100	25	100

Errors shown as raw counts and as the percentage of the total errors for that corpus.

**Table 4:** Examples for each error category.

error description	example
NER error	Rome tagged as location in ‘MacIntosh or Rome varieties’
text portion not extracted	location only appears in Acknowledgements
wrong/no geocode result	‘Moldova Region’ in Romania geocoded to Moldova country
comma group	‘Burlington, Cambridge’ taken as one location
candidate filtering error	company location not filtered out
non-standard headings	‘Almonds’ sub-heading contained study site info
other	wrongly extracted publisher location in footer

**Fig 4:**
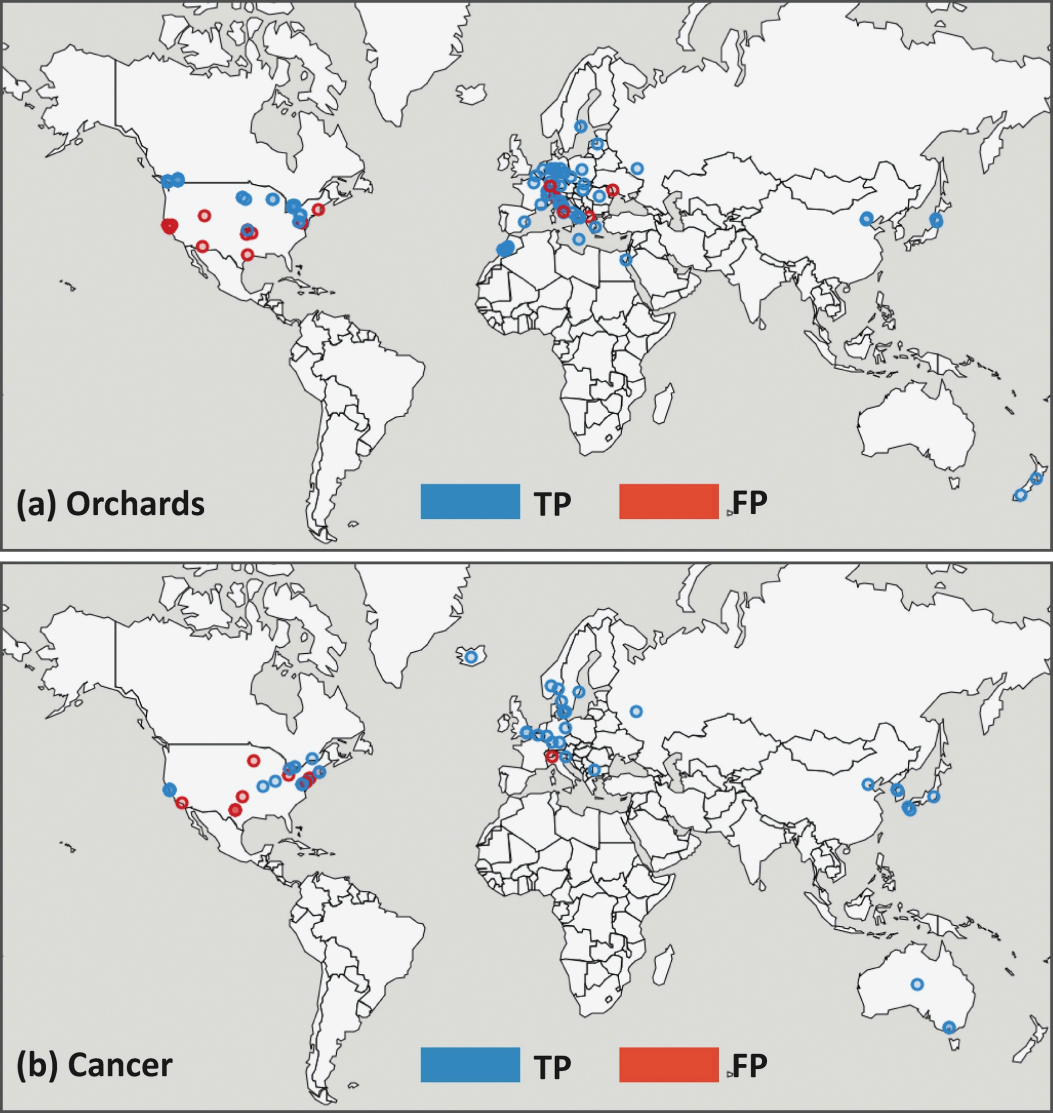
Global maps of geocoded locations. (a) Orchards corpus and (b) Cancer corpus. In both maps, full pipeline precision is represented, with true positives (TP) in blue and false positives (FP) in red. Note that any extracted string which gave a geocode result is mapped, whereas geocoding evaluation was done on true positive extracted strings only.

Fig 4 illustrates the spatial distribution of geocoded locations extracted from our two corpora at a global scale. Any extracted string which gave a geocode result is mapped, and hence the color-coding represents the full pipeline precision (c.f. full pipeline precision column found in Table 2). Note that, especially in the Cancer corpus, the majority of full pipeline false positives are due to wrongly extracted locations (extraction false positive), rather than geocoding errors. Hence the same map without color-coding would represent what one would see when mapping a new, unevaluated corpus. Both maps are dominated by locations in Europe and North America, demonstrating the underlying geographic properties of these corpora. For the Orchards corpus, locations around the Mediterranean reflect the underlying intent of the corpus. In the Cancer corpus, the locations identified suggest facilities capable of carrying out sophisticated genetic analysis of cancers. In both maps, false positives are predominately found in North America, likely reflecting both biases in the underlying spatial data used in geocoding and an underlying tendency of the geocoder to default to locations in North America.

## Discussion

In building our pipeline we made a number of assumptions and choices. Each of these has implications for the results, and we briefly discuss these here. Our first assumption was that the relevant locations were a subset of all location mentions in an article, and thus by filtering locations we would be able to improve performance. To quantify this assumption, we annotated all locations found in our test corpora to see how many would be evaluated as correct if extracted (strictly relevant locations, based on sentence context, are a small subset of these, as correct locations include for example author locations which happen to coincide with study sites). This is equivalent to using a perfectly performing off-the-shelf system to identify and geocode locations, and gives us a nominal baseline for our system. In this scenario, article unit precision for the Orchards subset is 0.628 and for the Cancer subset is 0.408, showing that relevant locations are a subset of all location mentions in both corpora, and less than half of all location mentions in the Cancer corpus. Assuming a recall of 1 for both corpora, this would mean *F*1 scores of 0.771 for Orchards and 0.579 for Cancer with a perfectly performing pipeline, were we to not target relevant locations. Our results are superior to these numbers, despite imperfections at each step of the pipeline, showing that targeting relevant locations is important.

An important question in building a pipeline such as ours is the overall influence of each step on our results. We carried out three experiments to explore the sensitivity of our results to individual steps. Our *Extract text* step uses knowledge about scientific domains to target portions of articles in which location mentions are more likely to be relevant. Turning off this step means that the whole article is processed in the pipeline (excluding references). The *Identify locations* step considers not only tokens classified as ‘locations’, but also those identified as ‘person’ and ‘organization’ to deal with noisy NER output and potentially improve recall. We simplified this step to consider only tokens tagged as locations (‘Locations only’). Finally, *Filter locations* uses a set of heuristics to increase overall precision, by discarding likely false positives from the retrieved location mentions. Turning off this step leaves all location mentions found in targeted article portions to be processed.

Table 5 shows the variation in *F*1 scores for each of these combinations. Precision, recall, and *F*1 are reported comprehensively in the S3 Table. in supplementary materials. For both corpora, turning off either the extraction step (‘No extract’) or the filtering step (‘No filter’) degraded performance. For the Cancer corpus, the performance is very clearly best when all pipeline steps are used. For the Orchards corpus, the difference is less marked, and indeed we note that with our ‘Locations only’ version of the pipeline, the location extraction performance is slightly better. These results demonstrate three key points. First, the text extraction and location filtering steps improve performance in all cases, reinforcing the importance and value of searching for locations in targeted article portions and retaining only relevant locations. Second, the sensitivity of the pipeline to the definition and processing of location tags is dependent on domain. The Orchards corpus refers to location in a straightforward way, and thus dealing with location in a more sophisticated manner (e.g. resolving locations related to organisations) does not improve performance. Third, these results demonstrate that our complete pipeline is necessary to handle documents from two very different scientific domains, with minimal customization. We suggest that this approach provides a good starting point to identify which steps should be further customized in new domains of scientific articles.

**Table 5:** F1 scores leaving out pipeline steps.

Corpus	No extract	Locations only	No filter	Full pipeline
Cancer	0.513	0.419	0.386	**0.754**
Orchards	0.606	**0.812**	0.719	0.802

Article unit location extraction F1 values for the 50 test articles per corpus. Best values are in bold.

Our pipeline also used, wherever possible, existing components, and through its modular design allows us to swap these relatively easily. To explore sensitivity of the pipeline to the geocoding API, we compared our geocoding results for true positive extracted locations with two commonly used and free alternatives (OSM Nominatim and the GeoNames API) on our test documents (Table 6).

**Table 6:** Comparison of geocoders.

geocoder	corpus	correct	incorrect	accuracy
Google	Cancer	50	1	0.980
OSM	Cancer	22	29	0.431
GeoNames	Cancer	19	32	0.373
Google	Orchards	99	10	0.908
OSM	Orchards	66	43	0.606
GeoNames	Orchards	62	47	0.569

Results for geocoding were evaluated using the subset of true positive extracted location strings, calculating how often the geocode result for these strings was correct or incorrect, giving a value for geocoding accuracy.

Since all three of these tools are treated as black boxes, we can only speculate as to the reasons for differences in performance. However, we believe the likely causes are ranking algorithms well suited to our use case, more sophisticated and effective string matching—especially for more complex location mentions—and more complete underlying gazetteers, in particular with respect to organisations (note that Google’s increased performance is markedly greater for the Cancer corpus where such matches are common).

In this work, our overall aim was to automatically extract and represent meaningful locations from scientific articles from both the ecology and biomedical domains. Relatively few works have been published on this specific problem and, of the works that share such an aim, the majority have focused on the ecological domain [12, 14, 15, 22, 24, 27], with two works examining a slightly broader set of journals still focused on environmental research [23, 27], one studying geology articles [25], and one in the hydrology/ hydrogeology domain [47]. In many of these works, location identification/extraction from text is performed manually [12, 15, 22, 23] or semi-automatically [14]. Our work shows that it is possible to build a fully automated pipeline, with limited customization across research domains within the broader text type of scientific articles, and obtain results of a high enough quality to be useful in the context of a meta-analysis or of a geographical search/filter for articles.

Comparing more broadly to state of the art work on evaluating location extraction from texts [48], our results are very encouraging. For two corpora, comparing five tools, Gritta reports precisions ranging from 0.21–0.81 for geotagging (equivalent to our extraction precision of 0.81 and 0.869 for the Cancer and Orchards corpora respectively). It is important to note that our pipeline is specifically designed for scientific articles, and thus not ‘out of the box’ as in Gritta’s experiments, but these results demonstrate that our approach is competitive with more specialized location identification systems.

Gritta [48] also notes that an important limitation of some current work is a tendency to develop customized tools for particular tasks and corpora [30, 31, 49]. We deliberately set out to build a more generic pipeline, whose focus lay on a task and a domain: identifying relevant locations from scientific articles using existing tools. Our approach therefore does not aim to optimize individual components of the pipeline (e.g. NER for toponym recognition or geocoding for toponym resolution), but rather aims to provide a useful set of filtered locations which can then be subject to human analysis. To facilitate this, we deliver locations in multiple formats (location strings, point coordinates, and location sentences), ready for review and correction by a human annotator to further increase overall precision, particularly using the location sentences. Confidence or uncertainty scores could also be assigned to each article, as is done in [50] where a baseline score is increased or decreased based on the intermediate outputs of a rule-based pipeline. Finally, our task and pipeline leads to output that is more manageable for a human annotator (e.g. in the context of a geographical corpus analysis), because we focus precisely on those locations that would be the main content locations for an article.

Although we aimed to develop a generic pipeline, we did include some elements of customization. In particular for the Orchards corpus, we attempt to extract more than location names by, for example, including cardinal direction terms. However, we make no attempt to extract truly compositional place descriptions such as ‘30km from Florence’ or interpreting these descriptions, though our code could be adapted to recognize these types of expressions and could be given to a system similar to the one in [50] used to georeference location descriptions for animal specimens. However, even if such expressions were extracted with high precision, current geocoding tools typically do not handle such expressions, despite long-standing calls to do so [37].

One important limitation of our work is the representation of all extracted locations as points. Although this is justified in most cases when mapping at a global scale (c.f. Fig 4), this may quickly become inappropriate depending on the properties of a particular corpus. Depending on our viewpoint and purpose, the Cancer corpus could be used to analyze locations related to the genetic analysis of tumour data (where point representations, related to specific facilities, are appropriate) or to explore locations related to tumour incidence (where more aggregated locations, related to large regions served by specialized hospitals, would be more meaningful). Although we are largely constrained to the use of points to initially represent all extracted locations, given points are returned by the geocoding service, we could also use a bounding box for a subset of results, which gives an indication of area. Furthermore, we could filter points by feature type, thus mapping only results of similar scales. Importantly, by keeping location representations in both textual and explicitly spatial form, there remains the possibility of re-generating and refining geometries using the extracted location strings.

Though a point is a rather simplistic way to represent a single scientific article, a larger collection of such points may be an appropriate way to represent and map an entire corpus of articles, particularly on the global scale where small differences in study site areas would not be visible. Global density maps, such as the kernel density map of sites in [15] or the rectangular-grid point aggregation in [27], can be created from point collections and are especially useful to highlight geographical research gaps in the corpus as a whole. As for interactive maps of study sites, a good example is JournalMap (https://www.journalmap.org/), a geosemantic search tool developed for an ecology-focused corpus where locations have been manually identified [22]. One straightforward enhancement of this tool would be to use bounding boxes to estimate the area/scale of study sites.

An alternative approach to performing our task would be to use sentence classification, including recently developed deep learning approaches [51]. Instead of identifying a set of relevant locations by targeting certain portions of the article (pre-NER) and filtering irrelevant locations (post-NER), one could instead classify each sentence in the article as either describing study/sample sites or not. Those sentences likely to contain study/sample site descriptions could then be further processed to extract location strings to be sent to a geocoding tool, such as is done in our work. Such a classification approach was used in [24] who classify sentences into ‘environmental’ or ‘experimental’ sentences, with the environmental sentences featuring relevant locations such as study sites, and experimental ones featuring irrelevant locations such as the provenance of chemicals.

## Conclusion

Writing a scientific paper is time-consuming and expensive, and we should maximize the value of each and every scientific work. Full-text analysis on large article collections is now possible, and should be increasingly applicable thanks to open access policies making more full-text articles available for processing. In this paper, we processed two collections of scientific articles, starting from collections of full-text PDFs, extracting locations using NER tools and rule-based processing, and geocoding these locations to spatially represent them.

Recording spatially explicit geographical information (such as a point coordinate, a bounding box, or a set of geometries) for scientific articles is an important step to facilitate meta-analyses and to identify geographical biases in scientific research. We tackled this problem by building an automatic processing pipeline, with the following takeaways:

- We use current tools, with minimal customization, making our pipeline easily extendable to other corpora of scientific articles.
- Our pipeline has high precision for identifying and resolving relevant location mentions (0.84 for an environmental corpus and 0.78 for a biomedical one) and is effective in extracting relevant locations at the article level (F1 0.81 for the environmental corpus and 0.75 for the biomedical one).
- We specify our task such that the aim is to filter and identify only relevant location mentions, suitable for both visualization and processing by human annotators. We reduce the number of location mentions to be triaged greatly through our approach.
- An error analysis reveals that failures can occur throughout the chain. These failures are also dependent on the nature of the problem specification (e.g. the difference between identifying all toponyms or identifying relevant location mentions).

An important limitation of our work lies in the use of customisation with respect to domain. Although we aimed to minimise customisation, introducing new domains of scientific articles may require further adjustment of the rules we applied. However, by minimising the extent of customisation, and identifying which components of our pipeline are most important, we believe that our approach could be transferred to new domains with respect to scientific publications. To do so, it is important that a pool of documents be annotated to explore how relevant location mentions are used in a text.

Future systems will benefit from improvements in the performance of individual system components (e.g. improved toponym recognition through deep learning approaches). Equally, the ability of geocoders to return more complex geometries, as appropriate for the scale of analysis, has clear potential for both representation and analysis of scientific corpora. We suggest that future work focus not only on such improvements in individual tasks, but also on gathering requirements from potential users of geographical exploration and search interfaces for scientific article corpora. The success of these approaches depends on their usefulness and practicality.

## Supporting information

S1 Appendix (PDF)

S1 Fig Location reporting quality over time for the Cancer corpus. We combined our location quality judgements with the manually annotated publishing years of all our manually annotated articles (*N* = 199, one article was excluded because it contained no samples and instead developed an algorithm) to plot the evolution of location quality reporting over time. For each time interval, we plotted the proportion of articles in that time interval which were in each of 4 location quality categories (good, medium, bad, none). The resulting plot suggests that location reporting quality is slowly improving over time. In particular, the proportion of articles reporting no location at all is steadily decreasing and the proportion of articles with either ‘good’ or ‘medium’ location reporting is trending upwards.
(PDF)

S1 Table Extended Orchards results. Result table including both results considering just studies (‘Orchards-studies’) and results for the full set of test articles (‘Orchards-full’). This full 50 document Orchards test set includes article types other than studies, including reviews, editorials, and popular science articles.
(PDF)

S2 Table Extended location unit results. Below, we show extended set of results for the location unit evaluation, which includes extraction recall and *F*1, and full pipeline recall and F1.
(PDF)

S3 Table Complete results for sensitivity tests. Evaluation of pipeline with test data set (50 documents per corpus).
(PDF)

S4 Table (a) Detailed annotation of 50 articles. Locations were annotated in 50 Cancer articles and classified into four categories: relevant, not relevant, correct if found but not strictly relevant (shortened as ‘correct’ in the table), and other. The counts for each category are shown in the table. (b) Detailed annotation of 50 articles. Locations were annotated in 49 orchard articles (after one duplicate was identified) and classified into four categories: relevant, not relevant, correct if found but not strictly relevant (shortened as ‘correct’ in the table), and other. The counts for each category are shown in the table.
(PDF)
