# Supplementary material for: Extracting and modeling geographic information from scientific articles
Source: PLoS One. 2021 Jan 6;16(1):e0244918. doi: 10.1371/journal.pone.0244918 (PMC7787447; doi:10.1371/journal.pone.0244918)
Supplement: S1 Appendix — (PDF) [file pone.0244918.s001.pdf]

S1 Appendix. **Article annotation.** We manually annotated 150 articles in total for the Orchards corpus and 200 for the Cancer corpus. For each article, the full contents were read, and when a portion of text indicated a relevant location (such as a study site location, or where patients were examined), the location string was copied to a new column, and the sentence context was also stored for reference. We stored further information in additional columns in this overall annotation, including the section heading where the relevant location appeared, a judgement on the quality of the textual location information, the year of publication of the article, the author locations, the number of authors, and whether the article featured a map. Many of the columns (such as number of authors) were not used further in the analysis.

For our test documents (50 articles per corpus), we annotated *all* locations appearing in each article, classifying each as 'relevant', 'not relevant', 'correct if found but not strictly relevant' (such as author locations which happen to correspond closely to the study site), and 'other' (such as borderline cases like 'European' in the expression 'European bat'). Additionally, for this comprehensive location annotation, two annotators annotated the same 5 articles in order to calculate inter-annotator agreement. Cohen's kappa for this overlapping set was calculated to be 0.903, showing a high degree of agreement between the annotators. The location annotation counts for these articles are presented in S4a Table and S4b Table.
