# Supplementary material for: Extracting and modeling geographic information from scientific articles
Source: PLoS One. 2021 Jan 6;16(1):e0244918. doi: 10.1371/journal.pone.0244918 (PMC7787447; doi:10.1371/journal.pone.0244918)
Supplement: S1 Fig — We combined our location quality judgements with the manually annotated publishing years of all our manually annotated articles (N = 199, one article was excluded because it contained no samples and instead developed an algorithm) to plot the evolution of location quality reporting over time. For each time interval, we plotted the proportion of articles in that time interval which were in each of 4 location quality categories (good, medium, bad, none). The resulting plot suggests that location reporting quality is slowly improving over time. In particular, the proportion of articles reporting no location at all is steadily decreasing and the proportion of articles with either ‘good’ or ‘medium’ location reporting is trending upwards. (PDF) [file pone.0244918.s002.pdf]

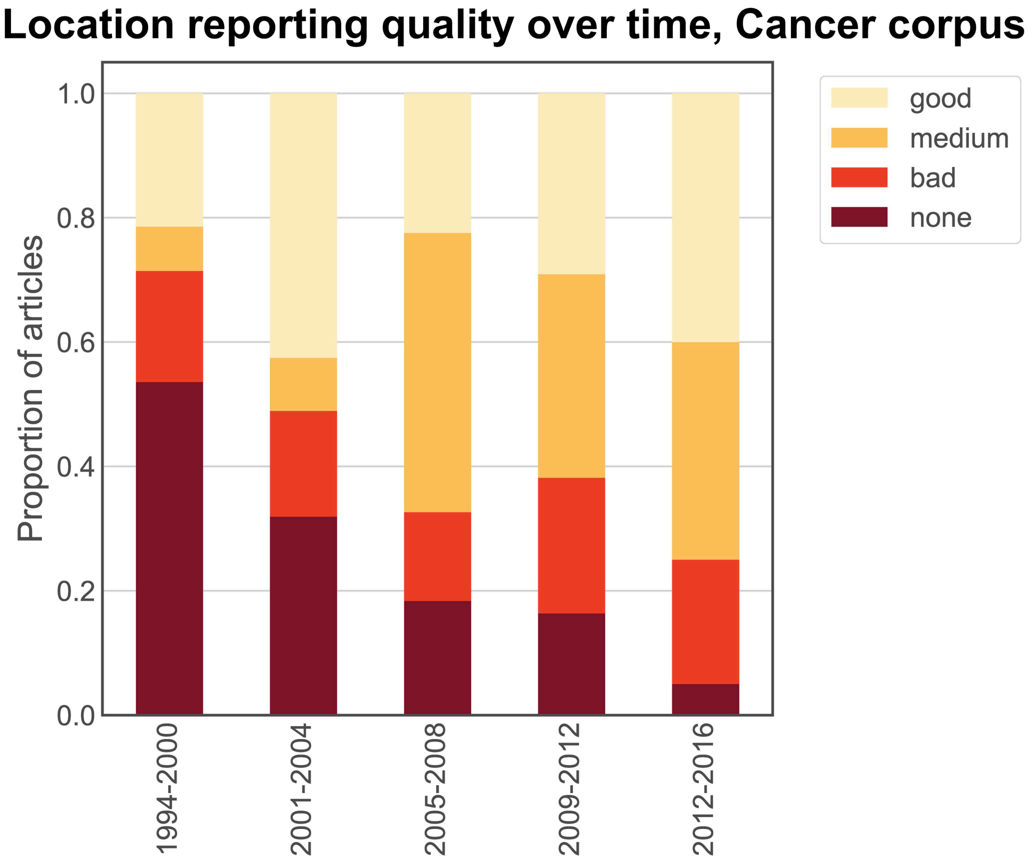

S1 Fig. Location reporting quality over time for the Cancer corpus. We combined our location quality judgements with the manually annotated publishing years of all our manually annotated articles ($N = 199$, one article was excluded because it contained no samples and instead developed an algorithm) to plot the evolution of location quality reporting over time. For each time interval, we plotted the proportion of articles in that time interval which were in each of 4 location quality categories (good, medium, bad, none). The resulting plot suggests that location reporting quality is slowly improving over time. In particular, the proportion of articles reporting no location at all is steadily decreasing and the proportion of articles with either 'good' or 'medium' location reporting is trending upwards.
