# Supplementary material for: Extracting and modeling geographic information from scientific articles
Source: PLoS One. 2021 Jan 6;16(1):e0244918. doi: 10.1371/journal.pone.0244918 (PMC7787447; doi:10.1371/journal.pone.0244918)
Supplement: S1 Table — Result table including both results considering just studies (‘Orchards-studies’) and results for the full set of test articles (‘Orchards-full’). This full 50 document Orchards test set includes article types other than studies, including reviews, editorials, and popular science articles. (PDF) [file pone.0244918.s003.pdf]

S1 Table. Extended Orchards results. Result table including both results considering just studies ('Orchards-studies') and results for the full set of test articles ('Orchards-full'). This full 50 document Orchards test set includes article types other than studies, including reviews, editorials, and popular science articles.

	location unit			article unit		
	extraction	geocoding	full pipeline	extraction (weighted)		
corpus	precision	accuracy	precision	precision	recall	F1
Orchards-studies	0.869	0.906	0.842	0.827	0.809	0.818
Orchards-full	0.872	0.908	0.846	0.841	0.766	0.802
