# Supplementary material for: Extracting and modeling geographic information from scientific articles
Source: PLoS One. 2021 Jan 6;16(1):e0244918. doi: 10.1371/journal.pone.0244918 (PMC7787447; doi:10.1371/journal.pone.0244918)
Supplement: S2 Table — Below, we show extended set of results for the location unit evaluation, which includes extraction recall and F1, and full pipeline recall and F1. (PDF) [file pone.0244918.s004.pdf]

S2 Table. Extended location unit results. Below, we show extended set of results for the location unit evaluation, which includes extraction recall and $F1$, and full pipeline recall and F1.

	location extraction			full pipeline		
corpus	precision	recall	F1	precision	recall	F1
Orchards-studies	0.869	0.828	0.882	0.842	0.787	0.814
Orchards-full	0.872	0.820	0.845	0.846	0.780	0.811
Cancer	0.810	0.823	0.816	0.778	0.817	0.797
