# Supplementary material for: Extracting and modeling geographic information from scientific articles
Source: PLoS One. 2021 Jan 6;16(1):e0244918. doi: 10.1371/journal.pone.0244918 (PMC7787447; doi:10.1371/journal.pone.0244918)
Supplement: S3 Table — Evaluation of pipeline with test data set (50 documents per corpus). (PDF) [file pone.0244918.s005.pdf]

S3 Table. Complete results for sensitivity tests. Evaluation of pipeline with test data set (50 documents per corpus).

corpus	experiment	precision	recall	F1
Cancer	No extract	0.374	0.814	0.513
Cancer	Locations only	0.428	0.410	0.419
Cancer	No filter	0.256	0.787	0.386
Cancer	Full	0.740	0.769	0.754
Orchards	No extract	0.460	0.887	0.606
Orchards	Locations only	0.852	0.777	0.812
Orchards	No filter	0.644	0.812	0.719
Orchards	Full	0.841	0.766	0.802
