# Supplementary material for: Extracting and modeling geographic information from scientific articles
Source: PLoS One. 2021 Jan 6;16(1):e0244918. doi: 10.1371/journal.pone.0244918 (PMC7787447; doi:10.1371/journal.pone.0244918)
Supplement: S4 Table — (a) Detailed annotation of 50 articles. Locations were annotated in 50 Cancer articles and classified into four categories: relevant, not relevant, correct if found but not strictly relevant (shortened as ‘correct’ in the table), and other. The counts for each category are shown in the table. (b) Detailed annotation of 50 articles. Locations were annotated in 49 orchard articles (after one duplicate was identified) and classified into four categories: relevant, not relevant, correct if found but not strictly relevant (shortened as ‘correct’ in the table), and other. The counts for each category are shown in the table. (PDF) [file pone.0244918.s006.pdf]

S4a Table. Detailed annotation of 50 articles. Locations were annotated in 50 Cancer articles and classified into four categories: relevant, not relevant, correct if found but not strictly relevant (shortened as 'correct' in the table), and other. The counts for each category are shown in the table.

Article title	relevant	not relevant	correct	other
Comparative genomic hybridization...	0	5	5	0
Genetic alterations in primary...	2	6	4	0
Breast cancer in young...	1	7	8	0
Genomic aberrations in pediatric...	2	6	10	2
The effect of chlorambucil...	0	11	0	0
Retained heterodisomy is...	1	7	5	1
Genomic, immunophenotypic...	0	2	5	0
Integrated genomic analysis...	2	13	5	0
Detection of genetic alterations...	1	2	6	3
Alteration of enhancer of...	1	5	4	0
Detection of chromosomal DNA...	0	18	0	1
Patterns of chromosomal alterations...	1	4	2	1
Comparison of chromosomal imbalances...	0	15	0	2
Genetic abnormalities detected by...	0	11	0	0
Novel genomic imbalances in...	0	5	4	0
CGH anaylsis of secondary...	0	7	1	0
Comparative genomic hybridization analysis...	0	5	6	4
High-resolution comparative genomic...	7	6	12	5
Loss of 1p and...	0	8	0	0
Chromosomal imbalances detected by...	0	5	2	1
Tumor necrosis factor...	1	26	4	1
Amplification and overexpression of...	2	18	11	0
U-2973, a novel B-cell...	1	21	4	1
Frequent genetic alterations in...	0	3	8	0
Gain in 1q is...	1	4	3	1
Characterization of a new...	0	11	2	0
Novel regions of chromosomal...	0	20	6	0
Genomic profiling identifies TITF...	1	22	1	1
Distribution and signifcance of...	2	6	6	1
Homozygous deletions and recurrent...	0	0	8	1
Genome-wide DNA copy number...	1	11	0	2
Copy number analysis indicates...	3	3	1	0
A SNP microarray and...	0	5	5	0
Multiplex Amplicon Quantification (MAQ)...	2	2	6	2
The cyclic AMP pathway...	4	7	13	0
Chromosomal and gene amplification...	1	1	2	0
Characteristic chromosomal imbalances in...	0	3	7	0
DNA copy number alterations...	3	3	4	0
Genomic markers for malignant...	2	20	10	0
Increased topoisomerase IIa expression...	2	18	5	2
Defining Genomic Alteration Boundaries...	0	15	2	0
High frequency of PTEN...	2	3	7	1
Deletions of Immunoglobulin heavy...	8	2	4	0
Recurrent deletion of 9q34...	1	9	8	0
Genomic and immunohistochemical analysis...	1	8	2	0
Oncogenic FAM131B-BRAF fusion resulting...	6	24	2	0
Identification of a HMGA2-EFCAB6...	0	9	3	0
Development of lung adenocarcinomas...	2	2	6	6
Somatic amplifications and deletions...	1	4	4	2
Variable extent of intra-tumor...	3	9	5	3

S4b Table. Detailed annotation of 50 articles. Locations were annotated in 49 orchard articles (after one duplicate was identified) and classified into four categories: relevant, not relevant, correct if found but not strictly relevant (shortened as 'correct' in the table), and other. The counts for each category are shown in the table.

Article title	relevant	not relevant	correct	other
Phytophagous mites and...	3	4	12	0
Temperature-dependent effect...	1	1	9	0
Flora and plant genetic...	25	104	82	15
Kaolin particle films...	1	9	4	3
Bats at risk?	5	8	4	7
Supporting crop pollinators...	2	3	11	3
Effects of plant diversity...	7	3	7	0
Effects of location, orchard...	39	8	13	5
Apple orchard pest control...	5	3	7	3
Species diversity and abundance...	33	6	10	2
An ecosystem services approach...	16	1	6	0
The use of extrafloral...	N/A	N/A	N/A	N/A
The use of benzyladenine...	N/A	N/A	N/A	N/A
Landscape enhancement of floral...	43	0	2	0
Risk to pollinators from...	N/A	N/A	N/A	N/A
Do age and type...	19	7	5	5
Flowers for better pest...	1	4	1	1
"Occurrence and distribution of...	36	24	5	2
The influence of study...	9	16	4	15
Habitat selection of three...	35	12	2	0
Farming and landscape management...	8	3	3	18
A survey of honey...	19	20	4	0
The use of insect...	69	1	3	3
Stable isotopes analysis to...	4	6	1	3
Do resources or natural...	6	1	2	1
Floral and Faunal Species...	6	0	1	0
Survey of natural enemies...	2	1	8	6
Increasing vineyard floral resources...	6	6	9	3
Irrigation of fruit trees...	0	6	0	0
Effects of Azinphos-Methyl on...	3	11	8	5
Interpreting orchardists' talk about...	6	4	17	0
Natural Enemies of the...	3	6	16	0
Unmown groundcover conserves adult...	3	0	6	1
Pollen removal and deposition...	2	4	0	10
An Assessment of DDT...	23	12	15	14
Plant species diversity for...	N/A	N/A	N/A	N/A
Thicket formation in abandoned...	8	4	6	0
Risk indicators affecting honeybee...	22	3	12	12
Cholinesterase inhibition in tree...	4	4	7	3
Exceptional cherry production in...	4	6	7	0
A New Survey of...	20	4	31	5
Integrated control of apple...	12	26	31	31
Insect taxa with similar...	3	0	4	0
Conservation ecology of bees:...	N/A	N/A	N/A	N/A
Flowering ground vegetation benefits...	10	16	9	6
The functional integrity of...	7	29	9	2
Determination of spinosad at...	4	15	0	2
Organochlorine Contaminants and Biomarker...	145	9	11	3
Richesse spécifique des ennemis...	N/A	N/A	N/A	N/A
